# Non-Recurrence Complications of Fibrin Glue Use in Pterygium Surgery: Prevention and Management

**DOI:** 10.2174/1874364101509010159

**Published:** 2015-11-04

**Authors:** Halil Hüseyin Cagatay, Gökçen Gökçe, Alper Mete, Yaran Koban, Metin Ekinci

**Affiliations:** 1 Department of Ophthalmology, Faculty of Medicine, Kafkas University, Kars, Turkey; 2 Department of Ophthalmology, Kayseri Military Hospital, Kayseri, Turkey; 3 Department of Ophthalmology, Faculty of Medicine, Gaziantep University, Gaziantep, Turkey

**Keywords:** Complication, Conjunctival autograft, Fibrin glue, Pterygium.

## Abstract

**Purpose:**

To present complications of using fibrin glue in conjunctival-limbal autografting in pterygium surgeries other than recurrences and discuss their prevention and management strategies.

**Materials and Methodology:**

The charts of all patients who underwent fibrin glue assisted pterygium excision surgery with conjunctival-limbal autograft transplantation from 2010 to 2013 were reviewed. Patients who developed complications except recurrence postoperatively were included in this study.

**Results:**

Sixteen (17.39%) of the 92 patients were detected with a complication. Graft dehiscence was diagnosed in 7 (7.6%) patients with 5 of them treated conservatively and 2 patients requiring suturing. Five (5.43%) patients were diagnosed with cyst formation between the graft and conjunctiva or in the graft-removal area; these cysts were primarily excised and no additional problems occurred. Corneal dellen developed in 3 (3.26%) patients and 2 of them regressed after cessation of topical steroids and application of lubricant therapy while one was treated with amniotic membrane transplantation. Residual fibrin glue particles had stiffened on the ocular surface, which resulted in intensive pain and irritation in one (1.08%) patient on the same day of the surgery. The patient’s complaints were reduced by removing these particles from the ocular surface under topical anesthesia.

**Conclusion:**

Complications in fibrin glue assisted pterygium surgery are relatively different from other techniques. To avoid potential complications of fibrin glue in pterygium surgery, peroperatively ophthalmologists should ensure the conjunctival autograft and conjunctiva are properly adhered, fibrin glue remnants are completely removed from the ocular surface, and no Tenon’s capsule remains between the graft and the conjunctiva.

## INTRODUCTION

A pterygium is a fibrovascular tissue that often originates from the nasal bulbar conjunctiva and extends onto the cornea in a wing shape. Ultraviolet radiation and dry, dusty and windy environmental conditions are known to play a role in disorders of the tear film layer. In addition, certain studies have emphasized that in etiology, the mutation of the P53 gene on chromosome 17 is involved [1, 2]. Although various methods of treatment are reported for pterygium, surgical intervention is the most commonly preferred treatment modality [3, 4]. Indications for surgery are pterygium tissue closure of the axis of sight, irregular astigmatism thereby causing visual impairment, significant and progressive growth toward visual axis, chronic irritation, recurring inflammation, ocular motility disorders and cosmetic reasons [3-5]. The aim of an ideal pterygium surgery is the prevention of recurrence, which is the most common complication. Intra-operative antimetabolites, conjunctival autograft transplantation and conjunctival limbal autograft (CLAG) transplantation and transplantation of amniotic membrane are the most commonly used methods to decrease the rate of recurrence after surgery [3-7]. In graft transplantation, fibrin glue shortens the operation time and enhances the post-operative comfort, compared with the use of sutures [8-1]. In this study, we discussed the prevention and management of post-operative complications other than recurrence in patients who underwent pterygium excision and CLAG transplantation using fibrin glue.

## MATERIALS AND METHODOLOGY

The medical records of all patients who underwent a pterygium excision surgery with CLAG transplantation using fibrin glue from 2010 to 2013 were reviewed. Sixteen (17.39%) of the 92 patients were detected with a complication except recurence of the pterygium in a minimum one year follow up time. The study was conducted in accordance with the tenets of the Declaration of Helsinki patients, with the approval of the local ethical review board. Systemic examination and complete ophthalmologic examinations were done for all cases. Patients with serious systemic illness, glaucoma, vitreoretinal disorder and ophthalmological diseases such as dry eye syndrome and pemphigoid were excluded from the study. The cases underwent pterygium excision followed by conjunctival autograft with limbal stem cells and all grafts were attached with fibrin glue (Tisseel, Two-Component fibrin Sealant Vapor Heated, Baxter Healthcare Corporation Glendale, CA, USA) as described by Ugurbas *et al. *[8]. All surgeries were performed by the same surgeon (HHC) under sub-Tenon’s anesthesia. Patients were administered topical antibiotics (0.3% Ofloxacin, Exocin^®^), steroids (0.10% Fluorometho-lone, FML^®^) and preservative-free artificial tear drops (Hydroxypropyl methylcellulose, Tears Naturale Free^®^) 4 times a day for 1 month. The patients were followed up at days 1, 7, 14, 30 and subsequently every 3 months for the first year, then annually thereafter. Cases were monitored for recurrence and complications. Any fibrovascular tissue development crossing the limbus onto the cornea was considered to be recurrence, because of its benign prognosis sub-graft hemorrhage was not considered as a complication and all complications except subconjunctival hemorrhage were recorded.

## RESULTS

The average age of 16 patients was 49.68±16.62 (range 29 to 81) years and mean follow-up time was 19.85±5.9 months (range 12-35 months). Clinical characteristics of the cases were shown in Table **1**.

**Fig. (1):**
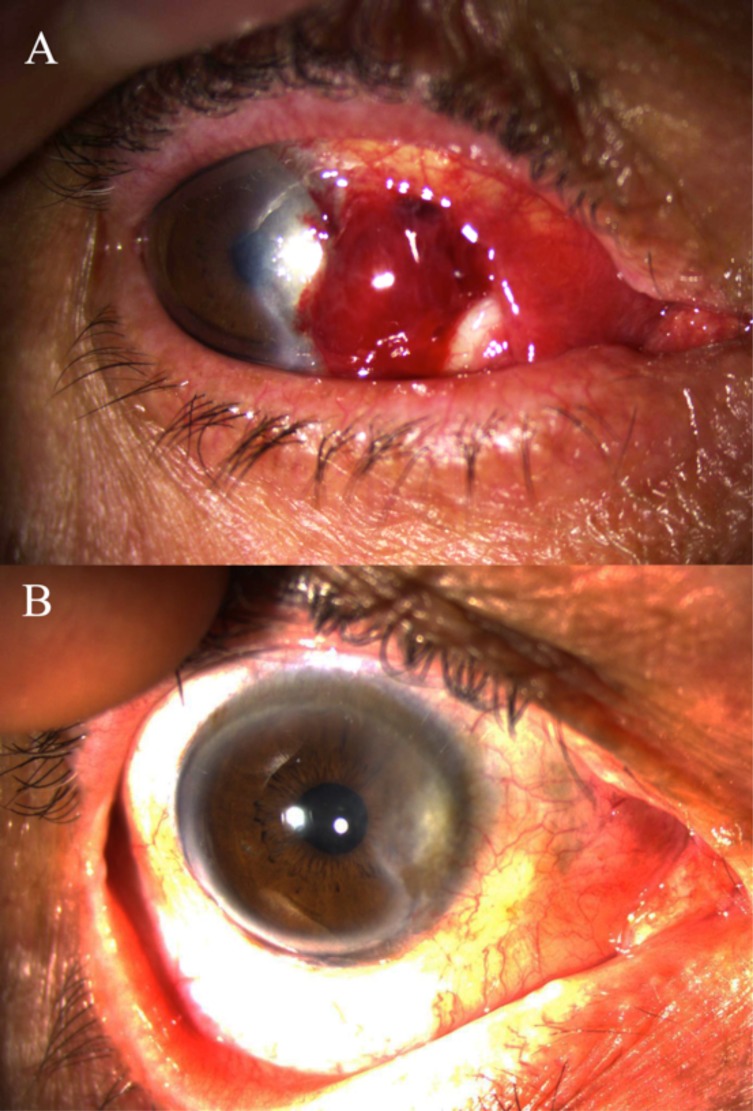
A: Early dehiscence in the nasal side of the graft and sub-graft hemorrhage under the graft. B: Conjunctival epithelialisation is provided with lubricant and occlusion therapy in the first week. Appearance is at second month after the surgery.

**Fig. (2):**
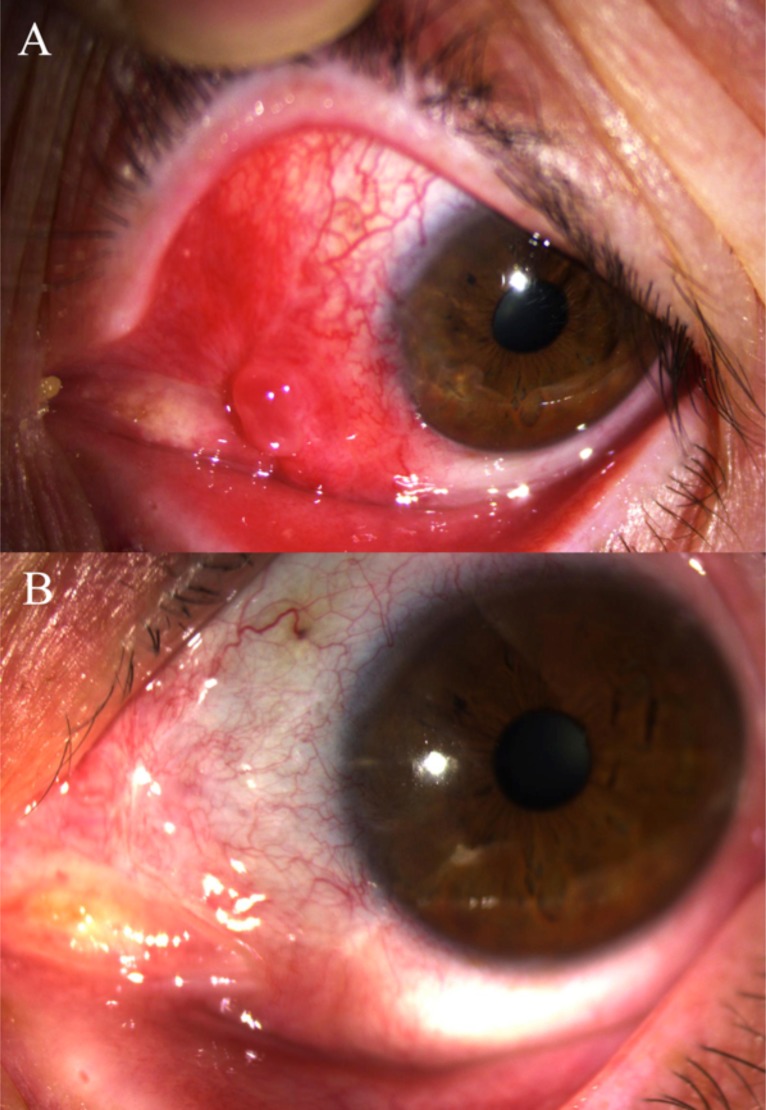
A: Cyst formation due to prolapsed Tenon’s capsule between the graft and conjunctiva. B: Cyst was primarily excised in the examination room and no additional problems occurred. Appearance is at sixth month after the surgery.

A total of 7 patients (7.6%) were diagnosed with early dehiscence in the nasal side of the graft, 5 of whom were treated with occlusion therapy and lubricant therapy, which provided conjunctival epithelialisation within one week. (Fig. **1**) Two (2.17%) patients required suturing due to a large size of the dehiscence. Five patients (5.43%) were diagnosed with cyst formation, 4 of them were detected between the graft and conjunctiva due to prolapsed Tenon’s capsule between the graft and conjunctiva and in one patient (%1.08), a conjunctival cyst developed in the donor area. These cysts were primarily excised in the examination room, and no additional problems occurred. (Fig. **2**) Corneal dellen developed in three patients (3.26%). Two of them regressed after stopping of topical steroids and continuing with only lubricant therapy and one was treated with amniotic membrane transplantation. One patient (1.08%) applied to the emergency service on the night of the surgery due to intensive pain and irritation. This patient was examined under topical anesthesia and it was observed that residual fibrin glue particles had stiffened on the ocular surface, causing irritation. The patient’s complaint was reduced after removing these particles from the eye surface under topical anesthesia.

## DISCUSSION

Various methods have been reported in pterygium surgery for reducing recurrence and complications, in order to obtain a smooth corneal surface and to achieve an easy and safe operation. Currently, the most common methods include the bare sclera technique, primary conjunctival closure, conjunctival autograft transplantation with or without limbal cells, surface reconstruction with amnion membrane and use of antimetabolites [3, 4, 12]. 

Recurrence rates of almost 80% have been reported for the bare sclera technique, which has a very short operating time [3]. It is often combined with the application of antimetabolites in order to reduce recurrence and serious complications have been reported from the use of intraoperative Mitomycin C (MMC) [3, 4, 12]. Onay *et al*. recently reviewed the records of 20 eyes of 18 patients who applied to their clinic for non-recurrent complications of pterygium surgery [12]. They indicated that bare sclera technique and intraoperative MMC application was associated with scleromalacia, dellen ulcer and perforation, whereas bad suturing was associated with pyogenic granuloma, inclusion cyst and the application of extreme cauterization with scleromalacia, symblepharon and dellen ulcer. 

Limbal conjunctival autograft and amnion membrane transplantation methods are used to reduce recurrence of pterygium and they produce fewer and milder complications than intra-operative antimetabolites and bare sclera technique [13-16]. Complication rates of amnion membrane transplantation are controversial [13, 17]. Ma *et al*., compared amnion membrane graft and conjunctival autograft combined with MMC treatment, and reported 1% pyogenic granuloma and 1% iatrogenic microhyphema as complications in the amnion membrane group, 1.8% scleral ischemia in the MMC group and 3.6% pyogenic granuloma and 7.3% conjunctival inclusion cyst in the conjunctival autograft group [13]. Besherati *et al*, compared the amniotic membrane transplantation with conjunctival autograft transplantation using 8-0 vicryl and reported an unacceptably high recurrence rate in amniotic membrane group [17]. Additionally they compared the non-recurrent complications and at 6^th^ month follow up visit, they detected non-recurrent complications including pyogenic granuloma in 4 patients (16.7%), graft dehiscence in 3 patients (12.5%) and conjunctival contraction in three patients (12.5%) in the conjunctival autograft transplantation group with a similar rate in the amniotic membrane transplantation group.

Although conjunctival autograft is a safe method that is also effective in the prevention of recurrence, fixing the autograft with sutures, prolongs operation time and creates complications relating to the sutures. These complications include increased conjunctival inflammation, giant papillary conjunctivitis, corneal ulcer, conjunctival granuloma and abscess. In addition, in cases where graft fixation is accomplished with sutures, events such as decreased patient comfort, symblepharon and graft laceration have been reported [18].

Koranyi *et al*., reported that the operation time had been shortened, postoperative pain was significantly lower, with no complications with using fibrin glue in conjunctival transplantation in pterygium surgery [19]. In a later study by Koranyi *et al*. on 461 eyes, they used fibrin glue in 325 eyes and vicryl suture in 136 [11]. Comparison of these two groups revealed that in the fibrin glue group, operation time was significantly lower and postoperative patient discomfort was less. The rate of recurrence was also lower in the fibrin glue group. Complications such as temporary graft edema and defects in the corneal epithelium were observed at equal rates in the two groups. Cagatay *et al*. compared fibrin glue to 8-0 vicryl suture and reported that fibrin glue group has favorable visual and refractive results with a similar complication rate [20]. In a study conducted by Yuksel *et al*., use of fibrin glue and 8/0 virgin silk suture for fixation of conjunctival autografts were compared [10]. They reported that the average operation time was significantly lower in the fibrin glue group, and postoperative symptoms were less common. Uğurbaş *et al*. applied fibrin glue to 10 patients [8]. In all cases except one in which the graft was kept small and thick, grafts were successfully glued into place and subconjunctival hemorrhage under the graft was observed in one patient. The average operation time was shortened in comparison to the suture group. Accordingly, they asserted that fibrin glue may be used instead of sutures in conjunctiva autografts as a method that shortens operation time and causes less discomfort in the postoperative period. Cha *et al*. compared 10.0 nylon suture (30 eyes) and fibrin glue (22 eyes); they did not observe any differences between these two materials in terms of frequency of complications [9]. In the group where fibrin glue was used, granuloma occurred more frequently (3 patients) and regressed upon medical therapy in two patients, whereas in one case it was treated with granuloma excision and amniotic membrane transplantation. No intervention was made in 7 cases in which early disengagement from nasal conjunctiva was observed; it was reported that these cases were epithelialized within one week.

We preferred to perform pterygium excision combining with CLAG transplantation using fibrin glue to avoid from the recurrences and disadvantages of sutures. In total we performed this technique in 92 patients and did not detect a serious sight-threatening problem at a minimum 1 year follow up time. Sixteen (17.39%) out of 92 patients were detected with a non-recurrence complication. Seven patients (7.61%) required additional simple surgical interventions such as conjunctival cyst excision and suturing, one patient (1.08%) required amniotic membrane transplantation while eight patients managed conservatively.

## CONCLUSION

Although fibrin glue is a material that shortens operation time, improves postoperative patient comfort and decreases inflammation, patients should be carefully followed in the postoperative time period for any possible complications. To avoid the postoperative complications of fibrin glue use in pterygium surgery, peroperatively ophthalmologists should ensure that the conjunctival autograft and conjunctiva are properly adhered, fibrin glue is completely cleared from the ocular surface, and no Tenon’s capsule remains between the graft and the conjunctiva.

## Figures and Tables

**Table 1.** Clinical characteristics of the cases.

Case No.	Age (Years)	Gender	Complication	Treatment
1	57	M	Dehiscence on nasal side	Lubricant + occlusion therapy
2	29	M	Dehiscence on nasal side	Lubricant + occlusion therapy
3	34	M	Dehiscence on nasal side	Suturing
4	75	F	Corneal dellen	Stopping of topical steroids + Lubricant therapy
5	65	F	Dehiscence on nasal side	Suturing
6	58	F	Corneal dellen	Stopping of topical steroids + Lubricant therapy
7	42	F	Cyst formation in donor area	Excision
8	46	M	Cyst formation between graft and conjunctiva	Excision
9	81	M	Corneal dellen	Amniotic membrane transplantation
10	75	M	Cyst formation between graft and conjunctiva	Excision
11	39	M	Cyst formation between graft and conjunctiva	Excision
12	39	M	Fibrin residue on ocular surface	Removal of fibrin residue from ocular surface
13	38	M	Dehiscence on nasal side	Lubricant + occlusion therapy
14	45	M	Dehiscence on nasal side	Lubricant + occlusion therapy
15	39	F	Cyst formation between graft and conjunctiva	Excision
16	33	M	Dehiscence on nasal side	Lubricant + occlusion therapy

M: Male, F: Female.
